# John Abercrombie, M.D.

**Published:** 1845-01

**Authors:** 


### OBITUARY.

### JOHN ABERCROMBIE, M.D.

It is our mournful duty to announce the sudden and premature decease of this great and good man. The sad event, which has deprived the profession of one of its brightest ornaments, and Edinburgh of one of her best citizens, took place at his residence in York Place, on Thursday the 14th of November, between the hours of nine and ten o'clock in the morning. About nine he finished a hearty breakfast; his carriage was waiting for him at the door, and some patients were likewise in attendance,—when, from his being unusually long in leaving his private room, his servant entered it, and found him lying prostrate and dead on the floor with his arms extended. From the position of his body, and from other circumstances, which we ascertained, but which it is not necessary to particularize, it would appear, that he had hurriedly advanced a few steps towards the door after feeling ill, and had then, when he found himself falling, extended his arms to save himself, but without doing so in any degree, as we infer from the nature and severity of a bruise upon the nose:—it was evident, in fact, that he had come to the ground a helpless and a lifeless weight.

Dr. John Abercrombie was born on the 10th October, 1781. His father was a respected clergyman of the Established Church of Scotland, in the city of Aberdeen. He graduated at the University of Edinburgh on the 4th of June, 1803, writing for his thesis, "De Fatuitate Alpina." We have often been told by one of his early friends,—one who was called as suddenly as he was from the shifting scenes of time,—that as a Student of Medicine, Dr. Abercrombie displayed that same deep, practical, unobtrusive piety, which in after life was the main-spring and regulating principle of his every action, the charm by which his talents were enabled to command the confidence of his patients and of the profession to an extent, if equalled, certainly never surpassed by a physician. After a short period of study in London, Dr. Abercrombie was admitted a Fellow of the Royal College of Surgeons of Edinburgh, upon the 12th of November 1804, upon which occasion he submitted to the President and Council of that body his 'Probationary Essay—on Paralysis of the Lower Extremities from Diseased Spine.' This paper is characterized by the same clear and terse style which is to be seen in all his subsequent writings. He now took a house in Nicholson street, and commenced practice as a general or family practitioner. At this period he devoted a great portion of his time to attendance on the poor, as one of the medical officers of the Royal Public Dispensary. In 1811, and subsequent years, when he had around him an effective body of ardent pupils, to whom he had succeeded in imparting much of his own enthusiastic passion for the careful clinical study of disease, he divided the city into districts, to each of which he attached several pupils. By his own zeal, and the hearty co-operation of his pupils, he thus became the instrument of much good to the poor; whilst at the same time he furnished himself with that rich field of observation which afterwards formed the basis of his future fame, eminence, and skill as a pathologist and practical physician. About this time he was appointed vaccinator along with Drs. Bryce and Gillespie; and with these eminent colleagues he took an active and efficient part in the introduction of the important discovery of Jenner.

In 1806, he published in the 12th volume of the 'Edinburgh Medical and Surgical Journal,' "A case of Cynanche Laryngea;" and in the Quarterly Report of the Royal Public Dispensary, which appears in the same volume, are to be found some remarks from his pen on "Tobacco injection in Dysuria." After this he continued for many years a frequent contributor to that celebrated miscellany; and, indeed, a large proportion of the cases which formed the basis of his works on the 'Pathology of the Brain,' and of the 'Abdominal Viscera,' originally appeared in its pages.

On the death of Dr. Gregory, in 1821, he became a candidate (along with Dr. John Thompson and others) for the chair of Medicine in the University of Edinburgh.* At that time, however, political feeling and professional influence of a kindred bias, were omnipotent in the municipal council of Edinburgh; and hence, neither Abercrombie nor Thompson was appointed.† It was at this time that Dr. Abercrombie gave up general practice, and became exclusively a consulting physician. To enable him to carry out this change with propriety, he became a Fellow of the Royal College of Physicians.

In his letter to the Lord Provost and magistrates, announcing himself as a candidate for the chair of Medicine, he gives the following list of his works:

1. On Diseases of the Spinal Marrow.
2. On Dropsy, particularly on some modifications of it, which are successfully treated by bloodletting.
3. On Chronic Inflammation of the Brain and its Membranes, including Researches on Hydrocephalus.
4. On Apoplexy.
5. On Palsy.
6. On Organic Diseases of the Brain.
7. On a Remarkable and Dangerous Affection, producing difficulty of breathing in infants.
8. On the Pathology of the Intestinal Canal. Part I, On Ileus.
9. Ditto. Part II, On Inflammation of the Bowels.
10. Ditto. Part III, On Diseases of the Mucous Membranes of the Bowels.
11. On the Pathology of Consumptive Diseases.
12. On Ischuria Renalis.

In 1824, appeared in the 'Medico-Chirurgical Transactions of Edinburgh,' two memoirs by him, the one "On the Pathology of the Heart," and the other on "The Nature and Origin of Tuberculous Diseases." His famous work on 'Diseases of the Brain and Spinal Cord' was published in 1828. This was followed by an enlarged edition of his work on the 'Intestinal Canal,' under the new title of a 'Treatise on the Diseases of the Abdominal Viscera.' His well-known work on the 'Intellectual Powers and the Investigation of Truth,' appeared in 1830; and in 1833, his 'Philosophy of the Moral Feelings.' About this time also he published a pamphlet entitled 'Suggestions on the Malignant Cholera,' and a short treatise on the 'Moral Condition of the Lower Classes in Edinburgh.' Besides the works which we have mentioned, he published several beautiful and highly useful Christian tracts, which were lately reprinted in one volume; and but a few weeks before his death he brought out what is perhaps the best of all his religious writings, Part First of his 'Elements of Sacred Truth for the Young.' We quote the concluding paragraph of the introduction to this little work, as well calculated to pourtray the goodness of the author's heart: "Should the work on which the author has thus entered be found useful as a manual, he will esteem it the highest distinction that can be conferred upon him. By the favour of the public, his former writings, on various subjects, have attained a most extensive circulation, and have received the most gratifying marks of approval. The ambition that now remains to him, is to have his name associated with those solemn and sacred hours, when the Christian parent calls around him the children of his heart, and, feeling all the uncertainty of life which is passing over them, seeks to raise their minds to a life that is never to end."

In 1834, the University of Oxford, in token of respect, conferred upon him the honour of its medical degree.

In 1835, he was elected Lord Rector of the Mareschal College of Aberdeen. The excellent inaugural address which he delivered to the students upon the occasion of his installation was extensively circulated at the time, and afterwards published under the name of 'Culture and Discipline of the Mind.'

On the accession of her present Majesty to the throne in 1837, Dr. Abercrombie was appointed First Physician to the Queen for Scotland; and at the period of his death he was the President of the Royal Society of Edinburgh.

In 1841, Dr. Abercrombie had a slight attack of paralysis, from which, however, he soon and completely recovered; and up to the moment of his death enjoyed good health. He was never known to complain of any symptoms indicating disease of the heart.

* His letter of application to the Town Council, which is now before us, is dated 19, York Place, 2d May, 1821.

† The death of Dr. Gregory occasioned several changes in the University. He was succeeded in the chair of Medicine by Dr. Home, then Professor of Materia Medica, and Dr. Home was replaced by Dr. Duncan, the Professor of Medical Jurisprudence, whose place, thus vacated, was supplied by Dr. Christison.

As has been already stated, Dr. Abercrombie died on the 14th of November. On Saturday the 16th an examination of the body was made by Mr. John Goodsir, in presence of various medical gentlemen.

*The encephalon* was in every respect healthy, with the exception of the arteries, which exhibited, in a very marked degree, the atheromatous deposit. There was no trace of old or recent effusion of blood. The internal carotids, at their entrance into the cranium were rigid, and somewhat dilated. None of the vessels were preternaturally distended. The ventricles contained a considerable quantity of serum ; and were of great size, corresponding to the enormous development of the encephalon, which weighed sixty-three ounces avoirdupois, being therefore one of the heaviest brains on record.*

*The pericardium and heart, &c.* On opening the pericardium, the heart was found enclosed in a mass of coagulated blood, which completely concealed it. On removing this clot, and raising the heart from the pericardium, a rupture, half an inch in length, was found on the posterior surface of the left ventricle, half-way between the left edge of the septum, and about midway between the base of the ventricle and the apex: the direction of the rupture was that of the axis of the ventricle; it did not extend into the cavity of the heart. The ruptured surface was ecchymotic; as was likewise, in its immediate neighbourhood, the substance of the ventricle itself, although covered by the pericardium. The heart was enlarged and dilated in a very slight degree ; it was considerably loaded with fat; and very soft in texture, as if from imperfect nutrition. The aorta presented a considerable quantity of atheromatous deposit. The valves were not altered in form, though they presented some atheromatous spots. The orifices of the coronary arteries were dilated; and throughout the heart, they had undergone the atheromatous degeneration; a branch of one of them passed down to the rupture, but its opening into the rupture could not be detected. A bristle was introduced into the ruptured orifice of a considerable coronary vein, and easily passed up towards the base of the heart.

The other viscera were healthy.

*Cause of death.* It appears then that the cause of death was a mechanical hindrance to the contractions of the heart, from effusion of blood into the pericardium by ruptured coronary arteries and veins. It is probable that the heart, softened from impaired nutrition dependent on the atheromatous state of its arteries, was torn, in the manner described, during its contraction.

FUNERAL. Although it was the wish of his family, that the funeral should be strictly private, the applications for leave to attend were so numerous as to render it necessary to extend the arrangements to a considerable degree. On leaving York place, the procession consisted of the hearse, his own empty carriage, eighteen mourning coaches, and a great many private carriages. On arriving at North st. Andrew street, the Free Church Presbytery of Edinburgh, and the Commission of the Free Church, joined the procession,† the members walking four deep, with Dr. Chalmers as Moderator of the Presbytery, and Mr. Grey as Moderator of the Commission, in the rear. On reaching No. 119 George street, (the temporary Hall of the College of Physicians,) the Royal College of Physicians, and the Royal College of Surgeons, amounting to about 150 gentlemen, met the funeral, and preceded it, four deep, the surgeons in front, having their president, Dr. James Simson, walking behind the members, and the physicians in like manner having their president, Dr. Robert Renton, walking in the rear. On reaching the gate of the West Churchyard, those public bodies opened up right and left, and stood uncovered, allowing the body, mourners, and friends, to pass between their ranks, after which they followed the procession to the grave in reversed order, having their respective

* According to the observations of Professor John Reid (Monthly Journal for 1843, p. 319,) the average weight of the encephalon of a man between fifty and sixty years is 50 ounces and 2 drachms. The two heaviest brains which we recollect are those of Cuvier and Dupuytren; the former of which was 63, and the latter 64 ounces.

† Dr. Abercrombie held the office of elder in Mr. Bruce's congregation, in connexion with the Free Church of Scotland.

office-bearers in front. In addition to those immediately attending the funeral, there were great crowds of the community, waiting the arrival of the procession throughout the streets, evincing the esteem in which the deceased was held by all classes. The ceremonial was unostentatious, and profoundly solemn.

Dr. Abercrombie's mind was eminently *practical*, a trait which shone forth conspicuously in all his writings, alike on medicine, morals, and religion. He scrutinised facts with singular jealousy; and we have heard it well remarked by a physician, that "what Abercrombie recognized as facts *must be facts*."

Dr. Abercrombie possessed every qualification required by the consulting physician—punctuality, an honorable and considerate bearing to his brethren, and a singular quickness of apprehending the nature of the case on which he was called to give an opinion. At the bed-side he was quite at home; there it was, that the depth of his penetrating glance, and the sagacity of his mind, were best seen and known. Those who were not acquainted with his manner, were often startled, on their first consultations with him, at the rapidity and accuracy of his diagnosis and prognosis. Although, doubtless, he completed a process of induction in his own mind, yet he was in the habit, unless particularly asked to do otherwise, to communicate only the result of his reasoning, and that in words as few and plain, as the English language could supply.

For the present, we will say no more of the great man who has left us; but upon some future occasion, when the present lamentations shall have subsided, it may be well—should no suitable pen in the interval take up the theme—for us to call the attention of our readers to a special consideration of his writings, the principal incidents of his life, and chief points of his character, especially as connected with the history and present state of medical science. J. R. C.

(*From Dr. Cormac's Journal*, Dec. 1844.)

---

